# Dietary supplementation with partially hydrolyzed guar gum helps improve constipation and gut dysbiosis symptoms and behavioral irritability in children with autism spectrum disorder

**DOI:** 10.3164/jcbn.18-105

**Published:** 2019-03-07

**Authors:** Ryo Inoue, Yuko Sakaue, Yuki Kawada, Ryuji Tamaki, Zenta Yasukawa, Makoto Ozeki, Satoko Ueba, Chihiro Sawai, Kazuo Nonomura, Takamitsu Tsukahara, Yuji Naito

**Affiliations:** 1 Laboratory of Animal Science, Department of Agriculture and Life Science, Kyoto Prefectural University, 1-5 Shimogamohangi-cho, Sakyo-ku, Kyoto 606-8522, Japan; 2 Department of Pediatrics, Shiga University of Medical Science, Seta Tsukinowa-cho, Otsu, Shiga 520-2192, Japan; 3 Taiyo Kagaku Co., Ltd., 1-3 Takaramachi, Yokkaichi, Mie 510-0844, Japan; 4 Moriyama Municipal Hospital, 4-14-1 Moriyama, Shiga 524-0022, Japan; 5 Kyoto Institute of Nutrition & Pathology, Ujitawara, Kyoto 610-0231, Japan; 6 Department of Molecular Gastroenterology and Hepatology, Kyoto Prefectural University of Medicine, Kamigyo-ku, Kyoto 602-8566, Japan

**Keywords:** gut microbiota, autism spectrum disorders, dietary fiber, constipation

## Abstract

Prebiotic dietary water-soluble fiber obtained from partially hydrolyzed guar gum was added to diets of children with autism spectrum disorders who presented constipation symptoms. Supplementation with partially hydrolyzed guar gum altered gut microbiota and significantly increased the frequency of defecation per week and altered the gut microbiota. In addition, supplementation with partially hydrolyzed guar gum significantly (*p*<0.05) decreased and tended to decrease (*p* = 0.07) the concentrations of serum interleukin-1β and tumor necrosis factor-α, respectively. More importantly, supplementation with partially hydrolyzed guar gum significantly ameliorated behavioral irritability as per the Aberrant Behavior Checklist, Japanese Version. The present study demonstrated that supplementation with partially hydrolyzed guar gum to diets of constipated autism spectrum disorders children helped improve constipation and gut dysbiosis symptoms, which in turn helped attenuate the level of serum inflammation cytokines and behavioral irritability.

## Introduction

Growing evidence has shown that children with autism spectrum disorders (ASD) present abnormal composition of the gut microbiota, a condition also known as gut dysbiosis.^(1)^ Gut dysbiosis putatively leads to systemic inflammation and neuroinflammation of the central nervous system that ultimately impairs brain functions (Gut-Brain Axis).^(2)^ Previously, we reported that in the fecal microbiota of Japanese ASD children, the abundance of bacterial genera *Faecalibacterium* and *Blautia* strongly correlated with the expression level of inflammatory genes in peripheral mononuclear blood cells (PBMCs). Moreover, the abundance of these two bacterial genera in Japanese ASD children was significantly different from that in typically developed (TD) children used as control.^(3)^ In addition, modulation of the gut microbiota via probiotic supplementation and diet intervention seems to help treat ASD effectively, as reviewed by Li *et al.*^(2)^ Thus, existing evidence strongly suggests that gut dysbiosis may be at least partially involved in the etiology of ASD and could be a potential therapeutic target.

Constipation is currently believed to be a causative factor of gut dysbiosis.^(4,5)^ Furthermore, constipation is known to be one of the major gastrointestinal disorders observed in children with ASD.^(6)^ For example, Ibrahim *et al.*^(6)^ found an incidence of constipation in 33.9% of 124 ASD children, which was significantly higher than that found in TD children used as control. Thus, the presence of constipation symptoms should be included as a critical factor when developing a treatment for ASD children.

Partially hydrolyzed guar gum (PHGG) is defined as a form of prebiotic dietary water-soluble fiber whose supplementation reportedly modulates the gut microbiota in healthy adults.^(7,8)^ More importantly, PHGG can be added without affecting the appearance or the flavor of diets and hence it is easy to give even to ASD children with exaggerated food preferences.

To the best of our knowledge, only one study has evaluated the effect of prebiotics on ASD children.^(9)^ Although Grimaldi *et al.*^(9)^ elegantly demonstrated that prebiotic galacto-oligosaccharides (GOS) altered the composition of gut microbiota in ASD children, anti-social behavior only improved when GOS supplementation was paired with food intervention.

In the present study, we aimed to evaluate the effect of prebiotic PHGG supplementation with no food intervention on constipation and gut dysbiosis symptoms and behavioral irritability in ASD children. To select ASD children as donors for the experiment, we set the presence of constipation symptoms as the critical criterion.

## Materials and Methods

### Ethical statement

The present study was registered in the UMIN Clinical Trial Registry (UMIN000034625) and conducted according to the Declaration of Helsinki. All procedures were approved by the Institutional Review Board of Shiga University of Medical Science (Approval Number: 27-161). Due to the limited comprehension of ASD children who acted as donors, written informed consent was obtained from their parents. Therefore, ASD children were unaware of PHGG supplementation. The privacy rights of ASD children were observed at all times.

### Subjects and study protocol

Thirteen outpatient children with ASD in the range of 4–9 years of age and living in similar geographic regions in Japan were enrolled in the present study (Table 1). The children were pre-categorized by the Diagnostic and Statistical Manual of Mental Disorders, 5th Edition (DSM-5) criteria^(10)^ and then diagnosed according to the Pervasive Developmental Disorders Autism Society Japan Rating Scale (PARS)^(11)^ and Modified Check-list for Autism in Toddlers (M-HAT).^(12)^ No medication (e.g., drugs or antibiotics) was administered to any of the children to treat either constipation or ASD for at least one month prior to the sampling period.

In the present study, none of the children underwent food intervention, but all of them were given PHGG supplementation (6 g/day; Taiyo Kagaku Co. Ltd., Mie, Japan). For easy ingestion, PHGG was dissolved in either food or a drink and given to ASD children during the period of regular examinations in the hospital, which lasted two months or longer (Table 1). The commercial PHGG concoction used in the present work was obtained from Taiyo Kagaku Co. Ltd. (Sunfiber; Yokkaichi, Japan). The PHGG concoction was prepared by treating guar gum with β-endogalactomannase produced by a strain of *Aspergillus niger*.

Parents of the 13 ASD children recorded their frequency of defecation weekly, from the week prior to the start of PHGG supplementation to the week prior to the end of PHGG supplementation. Moreover, feces were collected twice by the parents: the first collection was on the day immediately before the start of PHGG supplementation, and the second collection on the last day of PHGG supplementation. In addition, behavioral symptoms were evaluated by the Aberrant Behavior Checklist, Japanese Version (ABC-J) at roughly the same time as when feces were collected.^(13)^ ABC-J scoring was conducted by an *in situ* educator at the respective elementary school/kindergarten of the subjects. Tending to the special needs of the ASD children was among the core tasks of the score-taking educators, but they were unaware of the PHGG supplementation.

### Collection of feces and serum

Freshly evacuated feces were collected pre- and post-supplementation of PHGG using a stool collection brush and a storage tube (Wako Pure Chemicals, Osaka, Japan), which contained a DMSO-EDTA-salt solution (DESS) for the preservation of bacterial DNA.^(14)^ Peripheral blood was drawn pre- and post-supplementation of PHGG from nine of the children and collected in Venoject II vacuum blood collection tubes (serum collection tubes) (Terumo Medical Corp., Tokyo, Japan). Serum was collected by centrifuging the blood samples at 1,400 × *g*, room temperature for 10 min.

### Analysis of fecal microbiota by 16S rRNA metagenomics

Extraction of bacterial DNA from feces, library preparation, deep sequencing by MiSeq (Illumina, Tokyo, Japan) and data analysis were carried out exactly as described by Inoue *et al.*^(15)^ Data analysis was conducted using Biolinux,^(16)^ a Linux computing platform customized for bioinformatics research.

### Quantitation of serum cytokine and chemokine concentrations

The concentrations in serum of cytokines IFN-α, IL-1β, IL-6, IL-10, IL-12p70 and TNF-α and chemokines IP-10, I-TAC and MIG were simultaneously measured by a cytometric bead array (CBA; BD, Tokyo, Japan) using a flow cytometer (Accuri C6; BD). All procedures for quantitation were conducted as per the manufacturers’ instructions.

### Statistical analysis

α-Diversity indices Chao1 (richness) and Shannon (evenness) were calculated with the R phyloseq package.^(17)^ β-Diversity was estimated based on Bray-Curtis distances and tested by a principal coordinate analysis (PCoA) using phyloseq. The Bray-Curtis distance between the samples was statistically analyzed by a permutational multivariate analysis of variance (PERMANOVA). α-Diversity indices, the relative abundance (%) of bacterial genera, OTUs (operational taxonomic units) in the fecal microbiota, and the concentrations of cytokines and chemokines in pre- and post-PHGG supplementation samples were statistically compared using a paired Wilcoxson signed-rank test. Correlations between the frequency of defecation or the ABC-J irritability subscales and the relative OTU abundance were assessed by the Spearman’s rank correlation test using R software. Statistical significance was considered at *p*<0.05.

## Results

### Frequency of defecation and ABC-J scoring

 Defecation was observed only once per week in nine of the 13 ASD children and twice weekly in the remaining four ASD children prior to PHGG supplementation. During a week prior to the end of PHGG, it was observed that defecation increased from two times to four times per week in all children (Table 1; *p*<0.01). Significant differences were detected between pre- and post-PHGG supplementation as per the ABC-J irritability subscales. Indeed, the ABC-J irritability subscale was 15.1 ± 3.3 prior to PHGG supplementation, but decreased to 11.9 ± 1.9 after supplementing PHGG (Table 1; *p*<0.01). Nonetheless, other subscales of ABC-J remained unchanged despite PHGG supplementation (data not shown).

### Gut microbiota

**Fig. 1:**
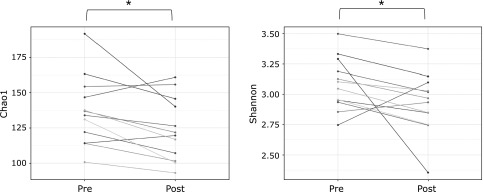
Chao1 and Shannon indices of fecal microbiota in 13 ASD children at pre- and post-PHGG supplementation periods. *p<0.05.

**Fig. 2:**
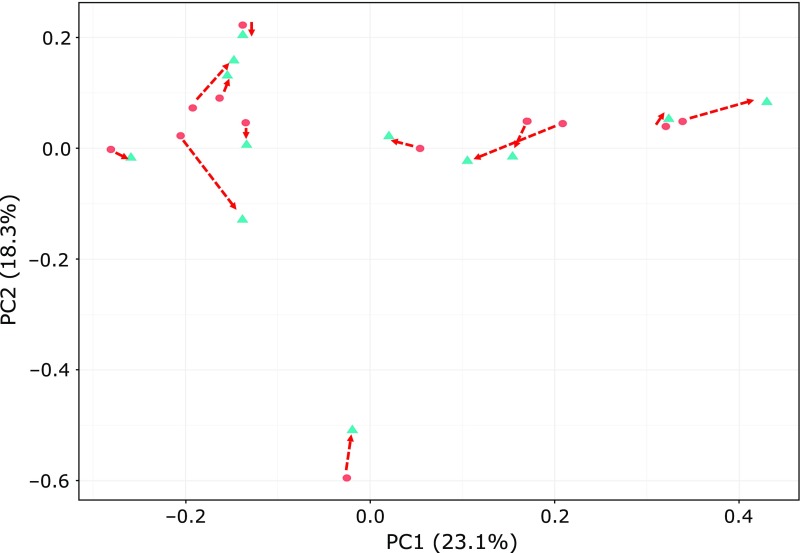
Principal component analysis plot of Bray-Curtis distance for the fecal microbiota of 13 ASD children at pre- and post-PHGG supplementation periods. Circles indicate data from the pre-PHGG supplementation period; triangles indicate data from the post-PHGG supplementation period. Red dotted arrows indicate individual changes in the coordinates of the plots from pre- and post-PHGG supplementation periods. PERMANNOVA; *p*>0.05.

**Fig. 3:**
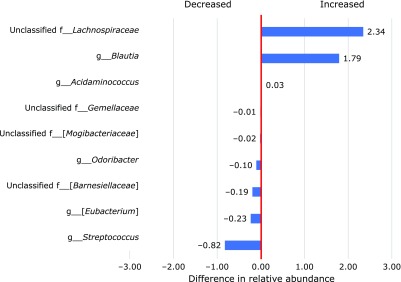
Comparative analysis of the taxonomic composition of the fecal microbiota at the genus level. Bacterial genera with significant differences between pre- and post-supplementation of PHGG are presented.

α-Diversity, both Chao1 and Shannon indices significantly decreased by PHGG supplementation (Fig. 1; *p*<0.05). The coordinates in the PCoA plots changed from pre- to post-PHGG supplementation for most PHGG-supplemented children, but β-diversity did not significantly differ between the data from the pre- and post-PHGG supplementation periods due to wide individual differences in the coordinates of PCoA prior to PHGG supplementation (Fig. 2). The relative abundance of nine bacterial genera changed significantly upon PHGG supplementation (Fig. 3). For example, the relative abundance of genera *Blautia* and *Acidaminococcus* increased significantly and that of genera *Streptococcus*, *Odoribacter* and *Eubacterium* (belonging to the family *Erysipelotrichaceae*) decreased significantly upon PHGG supplementation. However, four of nine genera significantly affected by PHGG supplementation were unclassified genera. As a result, it was decided to statistically compare the relative abundance of OTUs. PHGG supplementation significantly changed the relative abundance of 19 OTUs (Table 2). For example, PHGG supplementation significantly increased OTU_7, OTU_9 and OTU_52, which showed a high homology to *Fusicatenibacter saccharivorans*, *Blautia wexlerae* and *Acidaminococcus intestini*, respectively, but it significantly decreased 16 OTUs including OTU_68 showing a high homology to *Bacteroides ovatus*.

### Serum cytokines and chemokines

**Fig. 4:**
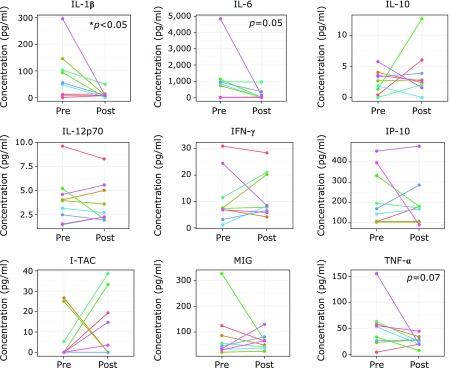
Concentrations of six cytokines and three chemokines in serum samples collected pre- and post-PHGG supplementation.

PHGG supplementation significantly decreased IL-1β (*p*<0.05) and tended to decrease IL-6 (*p* = 0.05) and TNF-α (*p* = 0.07), but did not affect other cytokines and chemokines (Fig. 4).

### Correlation of the OTU abundance with frequency of defecation and ABC-J irritability subscale

**Fig. 5:**
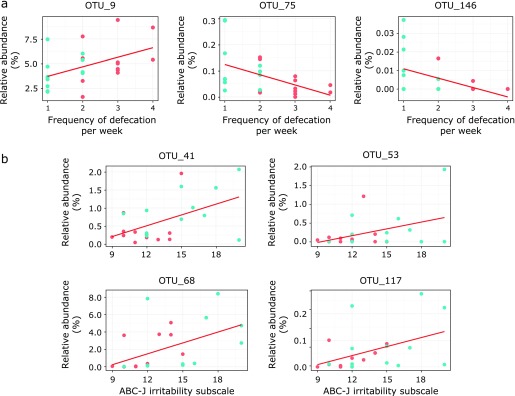
Correlation between the frequency of defecation and the ABC-J irritability subscales with the relative abundance of OTUs. Blue circles indicate the value at pre-PHGG supplementation; Red circles indicate the value at post-PHGG supplementation. Spearman’s R and *p* values are listed in Table 2.

While the relative abundance of OTU_9 showed a significant positive correlation with the frequency of defecation, the relative abundance of OTU_75 and OTU_146 correlated negatively with the frequency of defecation (*p*<0.05; Table 2 and Fig. 5). Finally, OTUs; OTU_41, OTU_53, OTU_68 and OTU_117 showed a significant positive correlation with the ABC-J irritability subscales (*p*<0.05; Table 2 and Fig. 5).

## Discussion

Recently, a greater number of ASD cases have been reported worldwide. According to a study by Idring *et al.* in Sweden,^(18)^ the prevalence of ASD in children aged 2–17 increased almost 3.5-fold from 2001 to 2011. The gut microbiota is one of the environmental factors involved in the ASD etiology and consequently, is attracting attention as a potential new therapeutic target. In the present study, we aimed to evaluate the effect of prebiotic PHGG supplementation with no food intervention on constipated ASD children.

Constipation symptoms in ASD children markedly improved after supplementation with PHGG (Table 1). The frequency of defecation in all 13 ASD children was less than 3 times per week prior to supplementation with PHGG and thus, the disorder was categorized as constipation as per the general definition.^(19)^ However, the frequency of defecation increased to more than 3 times per week in nine of the 13 ASD children after PHGG supplementation. In the remaining four children, the frequency of defecation was twice per week following PHGG supplementation, but since the original defecation frequency prior to PHGG supplementation was once per week in all four, it was considered that constipation symptoms improved moderately.

Our results showed that the gut microbiota was altered by PHGG supplementation. For instance, for α-diversity indices, both richness and evenness significantly decreased. In addition, PHGG supplementation changed the relative abundance of nine bacterial genera and 19 OTUs. It is possible that the aforementioned alteration of the gut microbiota contributed to improvement of constipation symptoms in ASD children. In past studies, alteration of the gut microbiota with supplementation with prebiotics or probiotics occurred simultaneously with improvement of constipation symptoms,^(20–22)^ but it was unclear what event preceded the other. Nonetheless, in a separate work, it was suggested that fecal microbiota transplantation, that is, artificial alteration of the gut microbiota, improved constipation symptoms.^(23)^ Moreover, in the present study, the abundance of OTU_9, OTU_75, OTU_146, which were significantly affected by PHGG supplementation, showed a significant correlation with the frequency of defecation (Table 2 and Fig. 5). Thus, both the existing evidence and our results seem to indicate that improvement of constipation symptoms is preceded by a beneficial alteration of the gut microbiota.

It is known that constipation and gut dysbiosis lead to an increase in mucosal permeability (leaky-gut)^(5)^ which may be also the case for ASD. Indeed, de Magistris *et al.*^(24)^ reported an increase in mucosal permeability in ASD children, and Emanuele *et al.*^(25)^ demonstrated an increased concentration of serum endotoxin in patients with severe ASD. Due to an increase in mucosal permeability, PBMCs in ASD children are likely to be more exposed to endotoxins than those in TD children, which causes PBMCs to be highly sensitized to endotoxin stimulation, as reported by Jyonouchi *et al.*^(26)^ In addition, Jyonouchi *et al.*^(26)^ detected that, as a response to endotoxins (lipopolysaccharides), PBMCs in children with ASD produced significantly higher amounts of IL-1β, IL-6 and TNF-α than did those in TD children. It is worth noting that in the present work, PHGG supplementation affected the same inflammatory cytokines. Indeed, it was found that in serum IL-1β significantly decreased and IL-6 and TNF-α tended to decrease. Taken together, it can be inferred that, by improving gut dysbiosis and leaky-gut symptoms, PHGG supplementation helped decrease the load of endotoxins from the intestine, which in turn resulted in a decreased production of serum inflammatory cytokines.

Brain inflammation is one causal factor for ASD and is believed to derive as a consequence of systemic inflammation and/or neuroinflammation of the central nervous system.^(27,28)^ Considering the brain-gut axis, systemic inflammation can be caused by a leaky gut and an elevated load of endotoxins, and neuroinflammation by inflammation of the enteric nervous system and the hypothalamic-pituitary-adrenal axis.^(2)^ Since PHGG supplementation was found to help attenuate systemic inflammation, it seems that PHGG supplementation, perhaps via improvement of the leaky gut, halts further progression of system inflammation. The obtained ABC-J irritability subscales seem to agree with these results, as behavioral symptoms of ASD significantly ameliorated after PHGG supplementation was initiated. It should be noted, however, that the frequency of defecation and ABC-J irritability subscales correlated differentially with the abundance of OTUs. For example, the frequency of defecation correlated with three OTUs and irritability subscales with four different OTUs. Therefore, this apparent discrepancy may imply that alteration of the gut microbiota by PHGG supplementation affected behavioral symptoms not only by improving constipation and leaky-gut symptoms, but also possibly by blocking neuroinflammation of the central nervous system.

Changes in the abundance of genus *Blautia* and OTU_9, which showed a high homology to *Blautia wexlerae* is of high interest. In our previous study, which was conducted in the same geographic regions,^(3)^ the abundance of genus *Blautia* was significantly lower in children with ASD than it was in TD children. Due to OTU_9 showed a positive correlation with the frequency of defecation, the decrease in *Blautia* genus detected in ASD children may be associated with constipation, which seems to provide evidence of the presence of gut dysbiosis, at least in Japanese ASD children. With respect to *Faecalibacterium*, in our previous study^(3)^ its abundance was higher in the gut microbiota of ASD children than in that of TD children, but in the present work it was unaffected by PHGG supplementation. However, in the present work the abundance of OTU_18, which showed a high homology to *Faecalibacterium praustitzii*, had a strong positive correlation with the ABC-J irritability subscales (*p*<0.05; Supplemental Fig. 1*). Therefore, based on these results, it is recommended that further research on the relationship of *Faecalibacterium* with ASD is conducted.

In summary, in the present work it was shown that PHGG supplementation to diets of constipated ASD children helped improve gut dysbiosis and constipation symptoms, which in turn helped attenuate the level of serum inflammation cytokines and behavioral irritability. Although the decreasing rate of the ABC-J irritability subscales may seem to be lower than that usually achieved by typical antipsychotics such as aripiprazole, but it should be stressed that this decrease was achieved not by medication, but merely by supplementation of prebiotics with no food intervention. Finally, in the present study, although the number of samples from ASD children may be considered relatively small, our results indicate that PHGG supplementation to diets may be a good therapeutic approach for treating ASD symptoms. A future clinical study using a larger number of samples is highly recommended as it shall provide more solid evidence and confirm our findings.

## Author Contributions

RI, YS, MO, TT and YN designed research, ZY and MO prepared PHGG, YS, SU, CS and KN enrolled the patients and collected fecal and serum samples, RI, YS, YK and RT conducted the experiments, RI, TT and YN wrote the manuscript. All authors have read and approved the final manuscript.

## Figures and Tables

**Table 1:** Profiles of enrolled ASD children

Variable	Pre-PHGG supplementation	Post-PHGG supplementation
Total *N* (male/female)	13 (12/1)	
*N* for feces and serum (male/female)	8 (7/1)	
*N* for only feces (male/female)	4 (4/0)	
*N* for only serum (male/female)	1 (1/0)	
Age (years)	5.9 ± 2.2	
Defecation frequency (/week)	1.3 ± 0.5	2.8 ± 0.7*
ABC irritability subscale	15.1 ± 3.3	11.8 ± 1.9*
Period of PHGG ingestion (months)	2–15 (median = 2)	

Defecation frequency and ABC irritablitity subscales were recorded from all participating ASD children. *p<0.01.

**Table 2:** List of OTUs that significantly differed between pre- and post-PHGG supplementation

OTU_ID	Nearest known bacterial species (BLANSTn)	Accession No.	Homology	Abuandance (%)				Correlation with defecation frequency			Correlation with ABC irritability subscale	
				Pre	Post	*p* value		R	*p* value		R	*p* value
OTU_6	*Bifidobacterium longum* strain HBUAS55049	MH685165.1	99%	2.19 ± 2.28	1.23 ± 1.03	0.01		–0.24	0.26		–0.19	0.38
OTU_7	*Fusicatenibacter saccharivorans* strain ASD2149	MG564316.1	99%	3.29 ± 3.05	5.38 ± 5.04	0.04		0.26	0.22		–0.03	0.90
OTU_9	*Blautia wexlerae* strain AUH-JLD17	KF374936.1	99%	4.14 ± 1.57	5.40 ± 2.23	0.03		**0.47**	0.02*		–0.25	0.25
OTU_22	*Bacteroides* sp. W20	LC033792.1	99%	1.33 ± 2.94	0.67 ± 1.48	<0.01		0.06	0.78		–0.23	0.28
OTU_24	*Phascolarctobacterium faecium *strain SN20	LN998073.1	99%	0.42 ± 0.86	0.31 ± 0.76	0.04		–0.26	0.21		0.22	0.30
OTU_41	*Streptococcus salivarius* strain NCTC7366	LS483366.1	99%	0.86 ± 0.62	0.41 ± 0.53	0.01		–0.33	0.11		**0.51**	0.01*
OTU_52	*Acidaminococcus intestini *strain DNF00404	KU726655.1	99%	0.15 ± 0.44	0.17 ± 0.50	0.047		–0.03	0.89		–0.38	0.06
OTU_53	*Alistipes putredinis *strain Marseille-P1137	LT223618.1	99%	0.33 ± 0.56	0.15 ± 0.34	0.02		–0.29	0.17		**0.41**	0.05*
OTU_54	*Oscillibacter* sp. PEA192	AP018532.1	99%	0.22 ± 0.19	0.13 ± 0.15	0.02		–0.07	0.75		–0.11	0.62
OTU_61	*Alistipes onderdonkii* partial strain Marseille-P591	LT558807.1	98%	0.19 ± 0.34	0.03 ± 0.05	0.03		–0.13	0.54		0.35	0.09
OTU_68	*Bacteroides ovatus *strain V975	LT622246.1	99%	2.56 ± 3.23	1.53 ± 1.92	0.03		–0.06	0.80		**0.49**	0.01*
OTU_75	[*Clostridium*] *leptum* strain DSM 753	NR_114789.1	95%	0.11 ± 0.09	0.05 ± 0.05	0.01		–**0.48**	0.02*		–0.05	0.82
OTU_86	[*Clostridium*] *innocuum* strain I46	CP022722.1	99%	0.15 ± 0.13	0.10 ± 0.16	0.04		–0.18	0.40		0.08	0.71
OTU_91	*Odoribacter splanchnicus* strain Marseille-P862	LT558826.1	99%	0.15 ± 0.23	0.05 ± 0.08	0.02		0.09	0.67		0.03	0.88
OTU_99	*Anaeromassilibacillus* sp. Marseille-P3371	LT725669.1	99%	0.03 ± 0.06	0.01 ± 0.02	0.02		–0.25	0.23		–0.09	0.69
OTU_102	*Clostridium* sp. 619	AB739698.1	98%	0.10 ± 0.15	0.07 ± 0.13	<0.01		–0.11	0.62		–0.06	0.77
OTU_117	[*Clostridium*] *indolis* strain BN-15	MF149975.1	99%	0.08 ± 0.10	0.03 ± 0.03	0.01		–0.24	0.26		**0.45**	0.03*
OTU_146	*Eubacterium* sp. Marseille-P3254	LT598590.1	97%	0.01 ± 0.01	<0.01	0.03		–**0.48**	0.02*		0.39	0.06
OTU_194	*Gemella sanguinis *strain NTUH_8428	AB775575.1	99%	0.01 ± 0.01	<0.01	0.03		–0.34	0.11		0.06	0.78

The Spearman’s correlation coefficient (R) and *p* value with significant correlation are indicated in bold letters and asterisk (*).
