# Supplementary material for: Dietary supplementation with partially hydrolyzed guar gum helps improve constipation and gut dysbiosis symptoms and behavioral irritability in children with autism spectrum disorder
Source: J Clin Biochem Nutr. 2019 Mar 7;64(3):217–23. doi: 10.3164/jcbn.18-105 (PMC6529696; doi:10.3164/jcbn.18-105)
Supplement: Supplemental Figure 1 [file jcbn18-105sf01.pdf]

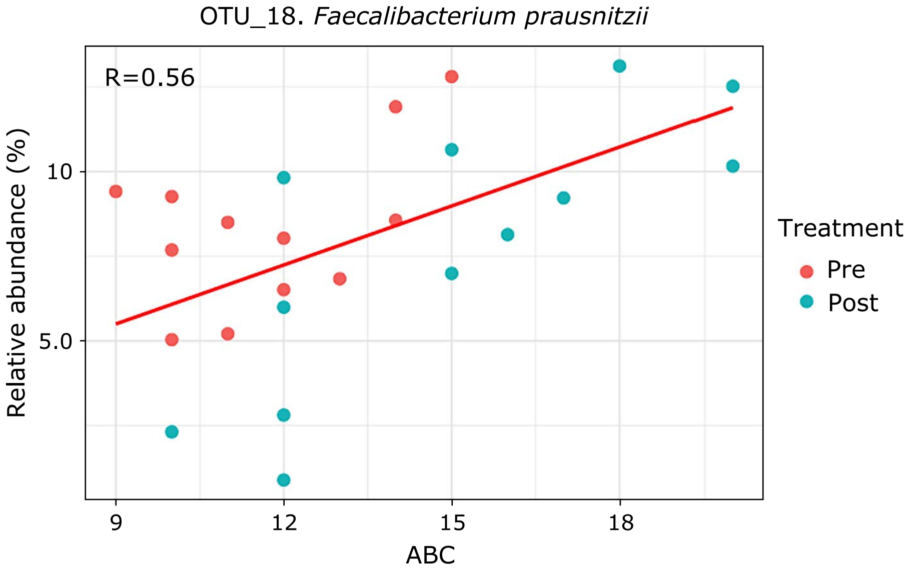

Supplemental Fig. 1. Significant correlation of ABC-J irritability subscales with the relative abundance of OTU_18, which showed a high homology to *Faecalibacterium prausnitzii*. $p<0.05$.
